# Information needs of patients undergoing bariatric surgery in Germany: a qualitative study

**DOI:** 10.1186/s12913-022-07950-2

**Published:** 2022-04-19

**Authors:** Jessica Breuing, Nadja Könsgen, Katharina Doni, Annika Lena Neuhaus, Dawid Pieper

**Affiliations:** 1 Institute for Research in Operative Medicine (IFOM), Department for Evidence Based Health Service Research, Faculty of Health, Department of Medicine, Herdecke University, WittenWitten, Germany; 2 Brandenburg Medical School Theodor Fontane, Institute for Health Services and Health System Research, Brandenburg, Germany; 3 Brandenburg Medical School Theodor Fontane, Center for Health Services Research, Brandenburg, Germany; 4 Faculty of Health Sciences Brandenburg, Brandenburg, Germany

**Keywords:** Information needs, Healthcare delivery, Bariatric surgery, Rouxen-Y gastric bypass, Sleeve gastrectomy, Qualitative interviews, Informed decision

## Abstract

**Background:**

Obesity is a worldwide problem with different treatment options. Bariatric surgery is an effective treatment for severe obesity; however, it leads to drastic changes (e.g., changes in everyday life and eating behavior) for patients, which may lead to information needs. Our aim was to identify the information needs of patients undergoing bariatric surgery and to explore the information provision within the healthcare process of bariatric surgery in Germany.

**Methods:**

We conducted a qualitative study (*n* = 14 single, semi-structured telephone interviews) between April 2018 and April 2019. The interview guide was designed prior to the interviews and consisted of four main sections (demographic information, pre-/postoperative healthcare provision, information needs). The interviews were transcribed verbatim and analyzed using qualitative content analysis with MAXQDA software.

**Results:**

There were unmet information needs with two factors (time: pre/postoperative and categories of information: general/specific) to be considered. Due to the patients’ description of information, we categorized information into general (different surgical procedures, general nutritional information) and specific (occurring simultaneously with a problem) information. Most patients felt well informed concerning general information. However, it was pointed out that it was not possible to provide complete information preoperatively, as the need for information only arises when there are postoperative (specific) problems. In addition, there seems to be a high demand for specific postoperative information regarding nutrition and nutrition-related problems. However, patients stated that postoperative nutritional counseling is not reimbursed by health insurance funds. The information conveyed in support groups and the exchange of experiences are highly valued by patients. However, some patients describe the information provided within the support groups as unfiltered, frightening or exaggerated.

**Conclusion:**

Overall, there were unmet information needs. Reimbursement by health insurance funds could increase the use of postoperative nutritional counseling and thus serve existing information needs. Support groups enable an exchange of experiences and therefore offer low-barrier access to information. Cooperation between support groups and healthcare professionals in information provision could be an approach to improving existing information needs or to avoiding the development of information gaps. Furthermore, the development and implementation of a digital solution for (postoperative) information dissemination could be helpful.

**Supplementary Information:**

The online version contains supplementary material available at 10.1186/s12913-022-07950-2.

## Introduction

Obesity is an increasing worldwide problem with different treatment options (lifestyle modifications or pharmacotherapy, and surgical interventions). Bariatric surgery (BS) has been successfully applied in the treatment of severe obesity. The use of BS as a treatment for obesity has been increasing in recent years. In 2013, the highest number of bariatric surgeries was performed in the USA/Canada, with 154,276 bariatric surgeries (44 procedures per 100,000 inhabitants) [1], in 2018 the total number of metabolic surgery and BS in the USA increased to 252,000 [2]. The majority of patients undergoing BS is female (80.7% of 810,999 cases between 2002 – 2011 in the USA [3]).-A total of 7,126 bariatric surgeries (8.8 procedures per 100,000 inhabitants) were performed in Germany in 2013. For reimbursement by (mandatory) statutory health insurance funds (HIFs), both the indication for BS (body mass index > 40 kg/m^2^ or > 35 kg/m^2^ with comorbidities such as type 2 diabetes mellitus or arterial hypertension) and participation in a defined weight management program (nutrition therapy, exercise and behavioral therapy over a period of 6 months) must be proven [4].

High health literacy seems to facilitate weight loss after BS [5]. Health literacy is described as “the degree to which individuals have the capacity to obtain, process, and understand basic health information and services needed to make appropriate health decisions” [6]. Therefore, providing health information could support patients in their decision-making process and may help to increase health literacy. Consequently, information provision in all areas that are affected by bariatric surgery (e.g., nutrition, dietary supplements, changes in drug use/dosage, and psychosocial life) is important. Malnutrition seems to be a problem in patients seeking BS as well as patients who have already undergone BS [7, 8]. As a result, post-BS patients are at risk of anemia due to insufficiency of several micronutrients [9]. There is decreased adherence to micronutrient supplements after BS, mainly due to the cost of dietary supplementation, difficulty swallowing dietary supplements or underestimation of the need for dietary supplementation [10]. Furthermore, patient education, especially through healthcare professionals, could improve supplement intake [11].

Bariatric surgery is the most effective therapy of obesity, but still represents a surgical intervention on a healthy organ. Furthermore, there is a variety of surgical procedures and ultimately two that are most commonly performed (sleeve gastrectomy and gastric bypass). Therefore, we assume that there is an increased need for information, since in addition to the question of whether an operation should be performed, the question of the type of procedure also arises. In addition, healthcare delivery as well as the provision of information is heterogeneous in Germany [12]. Hence, identifying the information needs relating to BS patients’ perspectives on information provision and related information needs is necessary.

The aim of this study was to identify the information needs of patients undergoing BS and explore the information provision within the healthcare process of BS in Germany.

## Methods

### Design

We previously published a study from the overall project "Information needs of patients undergoing bariatric surgery" [12]. In a previously published study, interviews were conducted with bariatric surgeons. The introduction of the present paper is based on the introduction of the previously published study. Because of the overlap of methods used (except e.g., recruitment), we adopted the methods used in the previously published study. We followed the guidance provided by the Text Recycling Research Project [13].

The study was approved by the Witten/Herdecke University Ethical Committee (224/2017). All methods were performed in accordance with the Declaration of Helsinki. Written informed consent was obtained from all participants.

This qualitative interview study is part of a larger research project to identify the information needs of patients undergoing BS. We designed a project with three qualitative interview studies (with patients, bariatric surgeons [12] and nutritionists) to identify the information needs of patients undergoing BS and map the information provision within the pre- and postoperative hospital process. The present study targets patients’ views on information needs and healthcare delivery in BS. Therefore, we used the same methods and description of our proceedings, so there may be various methodological overlaps between the papers. We defined information provision as “all processes involved in providing healthcare information to patients”. This includes the form of information (personal, e.g., in one-on-one appointments or in groups or written, e.g., as a flyer or webpage), timing of the information provision (pre/postoperative), and the information provided. Within the other interview study, we concentrated on bariatric surgeons and on information provision, healthcare delivery and information needs.

Choosing a qualitative approach was necessary because we assume that there is no standard in Germany regarding initiating the preoperative (information) process for patients. Therefore, preoperative healthcare provision, including information provision, had to be collected individually through qualitative interviews. One interviewer (JB), who is a nutritionist and doctoral candidate with a focus on BS and an experienced qualitative researcher, conducted the audio-recorded telephone interviews. There was no relationship between the interviewer (JB) and the participants. Participants did not know the interviewer; they only knew she was a researcher at Witten/Herdecke University.

We used the Consolidated criteria for reporting qualitative research (COREQ) checklist [14] to report our results (see Supplement 3).

### Recruitment

Eligible patients had to be 18 years or older and had to have had their first irreversible BS (sleeve gastrectomy or gastric bypasses) within the last 24 months in Germany. We chose a 24-month timeframe to decrease recall bias, except for the piloting. We included only irreversible BS patients because the consequences, especially weight loss, are more severe and the changes in body and lifestyle are permanent. For example, Roux-en-Y gastric bypass achieves significantly greater weight loss than laparoscopic adjustable gastric banding [15]. Patients were contacted with several approaches. We contacted all certified competence and reference centers for BS using a list of all certified centers. This list was prepared by the German Society for General and Visceral Surgery, which certifies these centers [16]. We contacted the centers by e-mail and asked them to hand out our patient recruitment flyer to eligible patients. Furthermore, we contacted several obesity and/or BS (patient organized) support groups and asked them to either hand out the patient recruitment flyer or post it on social media (e.g., Facebook). Additionally, we used the snowball sampling technique to ask participants after each interview if they had had any contact with any other eligible patients and, if so, we asked them if they could transfer our patient information flyer. There was no incentive for participation. The recruitment period started in April 2018 and ended in May 2019. Recruitment ended when saturation [17] was reached, which indicated that no new analytical theme emerged..

### Data collection

Data were collected from April 2018 to April 2019. The interview guide (Supplement 1: interview guide) was designed prior to the interviews and consists of four main sections (demographic information, preoperative healthcare provision, postoperative healthcare provision, and information needs) with predominantly open-end questions. It was reviewed and modified by an experienced nutritionist who worked in a clinic for BS in a university hospital for many years and was the head of their nutrition team. The first interview was used as a pretest, but it resulted in no modifications of the interview guide.

The interviews started with questions about the participant’s demographic information (sex, age, education, insurance status (statutory/private), type of surgical procedure, clinic where the operation was performed, duration since the operation, preoperative weight, current weight, and drug and nutritional supplement use). We categorized education into low (ISCED < 3), middle (ISCED 3/4) and high (ISCED > 5) based on the ISCED­2011­Level [18, 19]. Subsequently, the preoperative section dealt with questions about the decision-making process and healthcare processes (appointments with surgeons/nutritionists, support group meetings, and preoperative information provision). Then, the postoperative section continued with questions about healthcare processes (appointments with surgeons/nutritionists, support group meetings, and postoperative information provision), weight loss progress, dietary adaptions and changes in everyday life. The last section focused on information needs and future approaches for information provision.

### Data processing

The audio files of the semi-structured telephone interviews were transcribed verbatim by an external agency. Afterwards the transcripts were checked by the researcher. Participants were not involved in data processing nor analysis.

Based on the interview guideline, data codes were developed prior to the interview analysis by one researcher (JB) and checked by another (NK). The data codes were divided into nine groups:

1. Participants’ characteristics and general data
2. Preoperative care
3. Preoperative information
4. Costs
5. Postoperative care/information
6. Postoperative problems
7. Information needs
8. General Problems
9. Solutions

Furthermore, rules of coding (e.g., just one word or context) and code specifications were defined for each code and subcode (Supplement 2: data coding system).

### Data analysis

The transcribed interviews (including the pretest interview) were analyzed using qualitative content analysis [20] supported by MAXQDA software in order to structure the collected data into codes (categories) and subcodes (subcategories). The approach for developing codes was both deductive (the predetermined data codes, derivate by the interview guide) and inductive (elaboration of additional themes in the material as well as compile subcodes to the predetermined data codes). The deductive approach was used on document (whole transcript) level, while the inductive approach was used on code level to create subcodes. Two researchers (JB and NK) independently analyzed one-third of the interviews with the predetermined data codes. After discussion and consensus, the data codes were modified, and the given codes were adjusted. After achieving reasonable interrater reliability, further analysis of the remaining interviews was conducted by one researcher (JB).

## Results

We conducted *n* = 14 semi-structured interviews. There was no dropout or refusal of participation at any time. The duration of the interviews ranged from 21 to 70 min, with a mean time of 44 min. Participants had a mean age of 43.9 years, and most participants (*n* = 13 (92.9%)) were female. All participants took dietary supplements, and *n* = 5 (35.7%) had diabetes mellitus. Mean time since surgery was 11.8 months (SD 17.6). Participants had either sleeve gastrectomy (*n* = 8, 57.1%) or gastric bypass (*n* = 6, 42.9%) operations. The characteristics of individual participants are shown in Table 1.

**Table 1:** Characteristics of individual participants

	Age [years]	Sex	Education	Insurance status	Surgery	Weight before surgery/Current weight/weight change [kg]	Time since surgery [months]
P01	40–49	female	low	statutory	sleeve	254.4/144/110.4	72
P02	60–69	female	middle	statutory	bypass	112/76/36	6
P03	60–69	female	middle	statutory	bypass	155/98/57	18
P04	40–49	female	middle	statutory	bypass	126/76/50	7
P05	50–59	female	low	statutory	bypass	130/114/16	3
P06	40–4949	male	middle	private	sleeve	204/167.8/36.2	3.5
P07	50–59	female	low	statutory	sleeve	128/102/26	1.5
P08	40–49	female	middle	statutory	bypass	130/102/28	0.75
P09	40–49	female	low	statutory	sleeve	187/169.2/17.8	1.75
P10	20–29	female	middle	statutory	sleeve	154/79.5/74.5	15
P11	30–39	female	low	statutory	sleeve	138.6/115.5/23.1	3
P12	20–29	female	middle	statutory	bypass	143.5/97/46.5	9
P13	20–29	female	low	statutory	sleeve	145/109/36	16
P14	50–59	female	middle	statutory	sleeve	162.7/105/57.7	7

### Information provision

All patients had to see a surgeon and a nutritionist prior to surgery. During the individual appointments with the surgeon/nutritionist, most patients received information regarding different surgical procedures and their risks and cost reimbursement by HIFs. Most patients received information about postoperative diet and everyday life. Participant P01 stated that she refused to believe or tried to oppose the thought of the small size of the meals after the surgery and described herself as stubborn regarding preoperative information provision. P09 described a need for additional preoperative information regarding postoperative diet. Information regarding postoperative diet and everyday life was mainly provided by the nutritionist and rarely by the surgeon, and sometimes (additional) information was provided by the support groups.

### Information provision approaches

There were different information provision approaches. Healthcare professionals and support groups were the main sources of information. Additional sources of information included books and the internet. The internet was used to gain general information, such as information about different surgical procedures, as well as specific information through other patients’ experiences via social media, such as Facebook, blogs or a forum.

#### Healthcare professionals

In addition to the individual appointments with the surgeons/nutritionists (Table 2: Healthcare delivery), nine patients stated that they had additional written information (e.g., flyer, folder) provided by healthcare professionals. This written information could be either regarding medical or nutritional issues of the surgical procedures and/or bureaucratic procedures (e.g., reimbursement by the HIFs, applications).

**Table 2:** Healthcare delivery

	Median (IQR)	Minimum	Maximum
Number of appointments with the surgeon	2 (0,75)	1	4 to 5^1^
Duration of the first appointments with the surgeon [minutes]	25 (30)	5	60
Number of the individual appointments with the nutritionists (nutritional counseling)^2^	6 (3)	1	12
Duration of the first individual appointments with the nutritionists (nutritional counseling) [minutes]^1^	52.5 (26,25)	30	120

^1^One patient could not remember the exact number of appointments and gave a range of 4–5 appointments

^2^One patient did not receive individual nutritional counseling but did participate in a group session with other bariatric patients. They met 12 times for approximately 90 min

Patients stated preoperatively that they had various concerns and fears about BS. Nine patients felt that the healthcare professionals (nutritionist or surgeon) had listened to their issues and were able to help them overcome their fear.

Patients also expressed a desire for improvements regarding their information needs and healthcare delivery. For example, they requested postoperative nutritional counseling or more individual appointments with the nutritionist preoperatively.

#### Support groups

BS support groups in Germany are either online or local. Local support groups often cooperate with clinics, and clinics often provide premises for support group meetings. Sometimes cooperation with clinics implies regular visits by a bariatric surgeon and/or nutritionist. Other support groups are just organized by patients. The local support groups were either separated for pre- and postoperative patients or mixed.

Every patient reported the opportunity to join a support group either before and/or after surgery, and mostly all patients except P01 did so. The size of the support group seemed to intimidate P03 because the patient claimed to try it once, but there were 100 other patients and so she did not go again. P06 stated that there were two different local support groups (in different cities) and an additional support group in Turkish language that cooperated with the bariatric clinic. Most patients stated that they benefited from the exchange of experiences. P05 even chose her surgical procedure based on the experiences of other patients, which were exchanged at support group meetings. Furthermore, this patient highlighted the support group as a primary resource of information and indicated that talking to the surgeon to gain information was a barrier.

P05: “That [information provision by the support group] was very important for me. Because you could access it [the support group] again and again, even if you were in doubt, whereas you had some inhibitions as to bothering the doctor again and again.”

In general, the exchange with other patients either within a support group meeting or in private seemed to be essential for obtaining information and gaining trust in the information provided. The provision of information via support groups was labeled as “helpful” and “valuable” by the patients. An example of this is the assessment of pain after surgery or information about dietary supplements:

P05: “They [the healthcare professionals] did explain to me what happens afterward, that there can be pain and how it is the days after the surgery. However, as a whole, they [the patients] are all satisfied with it and have all lost weight well and are coping well with it.”

P09: “And all the statics of my body change due to this rapid decrease—quite clearly, the whole body changes. For example, you don't think about it beforehand, you don't know. That's what you learn in the support group. That's not bad. A support group is good. I also find it useful, for example, for information about which medications you take or which dietary supplements you take.”

Another suggested improvement was designating a sponsor/mentor picked from the support group who answers directly to his or her allocated protégé and could provide closer guidance.

### Information needs

There were unmet information needs with two factors to be considered. First, there are specific times when information needs could arise (pre/postoperative). Second, due to patients’ descriptions of information, we categorized information into general and specific information.

#### General and specific information

There seem to be two categories of information regarding BS. The first includes general information such as different surgical procedures and their risks, supplementation, general nutrition after surgery and bureaucratic procedures. Second, there is specific, problem-related information. The need for specific information only arises when there are postoperative problems. If patients needed specific information, this information was mainly provided within the support group by other patients. This second category of problem-related, specific information is mostly needed postoperatively and only if a problem occurs in a patient. At the end of the interview, patients were asked if they felt fully informed and how they would rate the information provision. Nine patients stated that they felt fully informed. This related predominantly to general information. Additionally, some patients pointed out that the information provision depends on individual factors, which can cause a need for specific information; therefore, the process of providing information cannot be claimed to be complete. P06 expressed stress regarding the provision of information because of the amount of new complex information, which needed to be processed retrospectively by the patient.

One factor that was often mentioned by the patients was the loss of satisfaction gained through eating (*n* = 4). In connection with this, patients feared eating in public because of the small amount of food they could eat. Other expressed concerns or fears were death and to the fate of their family if they died (*n* = 2), the inability to take care of children (*n* = 2), alopecia (*n* = 1), fear of weight regain (*n* = 1), marriage (*n* = 1), questions regarding the appropriateness of the decision (*n* = 1), and work (*n* = 1). Additionally, several patients just talked about fear in general without pointing out any specifics. P04 outlined the positive change regarding psychological issues (dealing with problems and thoughts of suicide) the surgery brought about in her. Preoperative psychological counseling was requested by P13.

Some patients claimed to have had further information needs or the need for additional healthcare (more preoperative nutritional counseling). There was a need for more detailed and specific information. Additionally, three patients expressed the need for psychological support after surgery.

#### Barriers to seeking information

##### I don´t want to bother the doctor

A barrier to patients seeking information seems to be the source of the information. Some patients indicated that they “*don´t want to bother the doctor*” (P05) or were more nervous speaking to the doctor than to the nutritionist (“*when you're sitting with the surgeon, you´re always more nervous than when you're sitting with a nutritionist*”, P09). Because of this, the nutritionist seems to be considered by patients as the party responsible for providing primary (preoperative) information.

##### *Costs*

Patients may face costs due to the following factors regarding BS: dietary supplements, plastic surgery (plastic surgery of the extremities or abdominoplasty), nutritional counseling and sometimes exercise courses within the preoperative weight management program.

There were several cost-related issues. Preoperative nutritional counseling (NC) generated costs in 8/14 patients. Costs to be borne by the patients ranged between 110 and 315€ for the entire nutritional counseling session. Of the patients who had to pay for NC, all but one knew that they had to pay the costs proportionally. In addition, costs for supplementation ranged from 0 (total reimbursement by the HIF) to 125€, with a mean of 27€ per month. The range of NC or dietary supplement reimbursement depends on the HIF. Most patients were aware of this but declared that there are dietary supplement products from different providers with a wide range of costs. The level of awareness regarding the process (including costs) for plastic surgery after BS was slightly different. Two participants (P10/P14) stated that they were not informed of the plastic surgery and its costs and reimbursement at all. The main sources for all cost-related information were the surgeons and the nutritionist, while some stated they received this information in the support group meetings.

## Discussion

Information provision seems to depend on many aspects—who provides the information, how the information should be provided, how specific the information should be, and at which point in the healthcare process the information should be provided. In addition, there is the question of how to assess and maximize the quality of this information.

### Healthcare professionals

The person delivering the health-related information on BS is an important factor. Healthcare professionals seem to be the primary source of trustworthy health information for patients [21], which supports patients’ understanding of their diagnosis, treatment decisions and possible prognosis [22]. Healthcare professionals, such as the bariatric surgeon or the nutritionist, were mentioned as a valid source of information by the interviewees, and they were able to take away patients´ fears. Talking to the doctor seems to involve a stronger barrier because the patients do not want to “bother” the doctor. Overall, there seem to be different barriers to the healthcare provided by physicians [23]. Participants described nutritionists as appearing to be closer to the patients and therefore presented themselves as the first professionals to address when information needs emerged. Preoperative NC is mandatory for covering the cost of BS by the statutory HIF but is itself either proportionally or not at all covered by the statutory HIFs. Postoperative NC is not covered by statutory HIF in most cases. Since postoperative NC is stated as essential in information provision, especially regarding specific information, reimbursement of postoperative NC may decrease information needs postoperatively.

### General and specific information

While many patients felt fully informed, there were some patients with unsettled information needs. Some patients said one could never be fully informed. Information provision regarding BS seems to depend on individual factors such as postoperative complications/problems. Information could be divided into general and specific information. There is general information that should be provided to every patient (such as different surgical procedures and their risks, and general nutritional information). Additionally, there is information that patients only seek or need if they have a specific, sometimes even rare, problem. Providing this special information to all patients could raise the issue of potential information overload that some patients already mentioned. Therefore, support groups seem to be a valid and important source of this specific information. A sponsorship (patient to patient) within the support group would provide closer contact and could therefore decrease barriers in information seeking.

### Information provision: the role of support groups and digital solutions

Information provision, emotional support and experience exchange were mentioned by the interviewees as key elements of support group meetings. Support groups have previously been shown to positively influence weight loss/maintenance through emotional support [24] or to support long-term weight loss more generally [25]. Additionally, either local or online support groups provide low-barrier access to information in comparison to clinical settings. Social media, such as Facebook, were used for online support groups. Facebook support groups seem to provide postoperative social support and are most effective if monitored by bariatric healthcare professionals who ensure the reliability of the information provided and screen for and correct inappropriate posts [26]. However, online support groups on social media could also provide medical or nutritional information without proper scientific citations, which can complicate information seeking for patients undergoing bariatric surgery [27]. Additionally, most bariatric patients integrate web-based information gathered through their own web searches in their decision-making processes [28]. Another possible approach providing specific information could be a digital solution such as an app. Digital solutions, such as online forums, could motivate patients regarding weight loss [29] but need to be supervised by a healthcare professional to avoid misinformation [30]. Likewise, local support groups could benefit from regular visits by a healthcare professional regarding the quality of the information provided. Therefore, web-based and local support groups present a possible communication strategy for providing high-quality health-related information on BS if they are monitored and/or edited by a healthcare professional.

## Limitations

A limitation of this study is the small sample size. However, we stopped recruiting in the event of suspected saturation. Additionally, there was an imbalance in sex (92.3% female), which may have led to bias although it reflects on the actual distribution in BS [3]. Nevertheless, we do not suspect an impact to our results because of this. A sex analysis was not conducted since it would not create valid results given high amount of female participants (92.3%).

Since the delivery of healthcare and the provision of information on BS in Germany is heterogeneous and depends on the clinic [12], the fact that 4/14 (28.6%) interviewees were operated on in the same hospital could lead to selection bias. All other patients underwent surgery at different hospitals. Since we report on information needs rather than healthcare structures, we do not suspect any impact to our results.

## Conclusion

Overall, there were unmet information needs. Support groups enable an exchange of experiences and offer low-barrier access to information. However, support groups would benefit from being monitored or supervised by healthcare professionals to improve the quality of the information provided and thus avoid misinformation. There seems to be a need for postoperative NC, which could be settled through reimbursement by the HIF. This could increase the use of postoperative NC and thus serve existing information needs. Cooperation between support groups and healthcare professionals regarding the provision of information could be an approach to improve existing information needs or to avoid the development of information gaps. Furthermore, the development and implementation of a digital solution, such as an app or digital support group, for information dissemination could be helpful, especially postoperatively.

## Supplementary Information

**Additional file 1:** **Supplement 1**. interview guide patients.

**Additional file 2:** **Supplement 2.** Coding system patients.

**Additional file 3:** **Supplement 3.** COREQ-Checklist patients.

## Data Availability

The datasets generated and analyzed during the current study are not publicly available because of ethical concerns regarding the privacy and confidentiality of participants. The datasets, however, are available from the corresponding author upon reasonable request.

## Abbreviations

BS: Bariatric surgery
FAQ: Frequently asked questions
HIF: Health insurance fund
NC: Nutritional counseling
