# Supplementary material for: Information needs of patients undergoing bariatric surgery in Germany: a qualitative study
Source: BMC Health Serv Res. 2022 Apr 19;22:515. doi: 10.1186/s12913-022-07950-2 (PMC9017015; doi:10.1186/s12913-022-07950-2)
Supplement: Supplementary file 1 — Additional file 1: Supplement 1. interview guide patients. [file 12913_2022_7950_MOESM1_ESM.docx]

The original German version of the interview guide has been translated with DeepL (https://www.deepl.com/translator).

**Interview guide – bariatric patients**

**Introduction**

Hello my name is Jessica Breuing from the Institute for Research in Operative Medicine at the Witten/Herdecke University in Cologne. We have been in contact before. I am very pleased that you support our study "Information needs of bariatric surgery patients" by participating in this interview.

You were already informed about the purpose of this study by the information sheet received by email. I will now ask you a few questions, which I would like you to answer freely. There are no right or wrong answers; I just want to hear your opinion or experience on various topics. We will slowly move forward with the questions from the beginning, that is, the first steps before surgery, to the time after surgery. Please get in touch at any time if you don't understand a question in terms of content or acoustics. Do you have any questions in advance?

Do you agree to my recording the interview? This will help us with the subsequent transcription and evaluation.

You have already read in the privacy statement that your data will be used exclusively for this current project and only in pseudonymous form. This means that a code will be assigned to your name. Therefore, the interview results will be published without being assigned to your name. Do you agree?

Okay, then we can start. (Switch on the recording device!)

I would like to start by asking a few things that relate specifically to you as a person as well as your operation.

- I note once your sex, you are (female/male). Would you agree?
- Would you please tell me your age?
- Would you tell me the highest level of education you have achieved?
- Do you have statutory or private health insurance?
- Which surgical procedure did you have? (wait) A sleeve gastrectomy, a gastric bypass or a combination procedure (if yes-inquire).
- Where was the operation performed? (If only the city is mentioned, please ask for the clinic).
- How long has it been since the operation?
- Would you tell me your weight before the operation?
  - Are you satisfied with the weight loss so far?
- Due to the [name surgery procedure], do you need to take any medications, e.g., dietary supplements, or be injected?

IF YES

- - Will you incur any costs as a result?

IF YES

- - - Were you aware of this beforehand?

**Pre-OP**

I would now like to talk to you about the time before the operation. Let's start at the beginning.

- How did you get the idea to undergo [name surgical procedure]?
- You just said that the surgery was performed at the [above-mentioned clinic]. How did you come up with [clinic]?
- Have you talked to friends/family about the planned surgery?

IF YES (if not already mentioned)

- - At what point did you talk to friends/family about the surgery? E.g., before the actual decision to have surgery or after the decision was made.
- Did you go to the doctor first, e.g., primary care physician or surgeon or dietitian, with the desire to have surgery? (If not already mentioned, ask which specialist the doctor was)
- Were you informed about the requirements for the reimbursement of costs of the operation by the health insurance fund? (Explain if necessary: For example, the psychological assessment or the visit to the nutritionist)
  - Who informed you about this?
  - How much time was there between the application and the consent for the operation?

I will now ask questions about the exact procedure before the operation. I will ask these in two blocks. First, we'll talk about the conversations or appointments with your surgeon, then I'd like to know more about the conversations with your nutritionist.

Now think about the first conversation with your surgeon. Can you give me a brief description of how that conversation went and what was discussed there?

(if not already mentioned)

- How many times did you see your surgeon prior to surgery, and did he or she perform the surgery?
  - How long did these appointments last on average?
- Were the various surgical procedures discussed?

(if not already mentioned)

- - Were the advantages and disadvantages of each procedure explained?
  - I.e. did you also talk about risks and consequences of the individual procedures?
  - Did you also talk about everyday life after the operation? (if applicable: i.e. how your diet will change).
  - Were you informed about possible costs arising from the operation? I mean here something like costs for dietary supplements or possibly costs for plastic surgery.

IF NO

- - When were the various procedures first discussed?
  - Was there a separate educational discussion?
- What information did you receive about the time after the operation?

Now, when the decision for [name surgical procedure] was made, was this a decision you made independently or were you guided by the surgeon's recommendation?

IF DECIDED INDEPENDENTLY

- Why exactly did you choose this procedure?
- What were the decisive reasons to have such an operation performed after you knew all the risks?

Now I would like to talk to you about your first preoperative contact with the nutritionist. Please tell me when you first saw a nutritionist and what the content of that conversation was?

(if not already mentioned)

- Did you try to lose weight with the support of the nutritionist before you considered having surgery?
- How often did you see the nutritionist before surgery?
- -How long did the appointments with the nutritionist last on average?
- -Did you have to pay for the nutritional counselling yourself?

IF YES

- - Were you aware of this beforehand?

Now please think about the time when the decision to operate as well as the surgical procedure was made. What was the role of the nutritionist after this decision was made?

(if not already mentioned)

- Did you still had appointments with the nutritionist afterwards?

IF YES

- - Was there also some kind of educational discussion there?
  - Was there any discussion about dietary changes before and after surgery?

IF YES

- - - What exactly was discussed about the change of diet?

Now I have some general questions about the time before the surgery. Before the operation, did you talk to other patients who had already had such an operation?

- What experiences were shared with you?
- Was the experience exchange helpful, i.e., did you still get information you hadn't gotten from the doctor or nutritionist?

IF YES

- - What additional information did you receive as a result?

Have you obtained additional information through other sources such as the Internet or your health insurance company?

IF YES

- (if not already mentioned) What sources did you use?
- What information did you seek through this source(s)?
- Were there any questions that you did not get an answer to through this source(s)? IF YES
  - What questions were these?
  - How did you proceed from there?

Now, if you think about the conversations you had with both the surgeon and the nutritionist, did you often ask questions there?

IF YES

- Can you remember some of these questions?
- To whom did you ask these questions?
- Were they questions that served to deepen the information you had already received? Or did you ask questions about topics that had not been addressed before?
- Did you express any fears or concerns in these conversations?
  - What exactly did you fear or worry about?
  - Did you express this to the surgeon or nutritionist?

**Post-OP**

Now that we've talked about what happened in the period before the [name operation], I'd like to talk to you about the period after the [name operation].

- What changes related to diet and eating behavior have occurred after surgery?
  - Were you aware of these changes beforehand?

IF YES

- - - Who informed you about these changes?

IF NO

- - - Would you have liked to have had this information beforehand?

IF YES

- - - - Who should have given you this information?
- What changes related to daily life have occurred after the operation?
  - Were you aware of these changes beforehand?

IF YES

- - - Who informed you about these changes?

IF NO

- - - Would you have liked to have had this information beforehand?

IF YES

- - - - Who should have given you this information?
- (if not already mentioned)
  - Have you shared experiences with other patients?
  - How helpful do you consider the exchange with other patients?

What information did you receive before [name surgery] that you found most helpful for the time after the surgery?

- (if not already mentioned)
  - Who gave you this information?

IF SELF-SOURCED (e.g., internet/other patients/support group).

- - - Who would you say should have gave you this information retrospectively?
      - Can you give me a reason for this?

Would you say that they were informed about all the changes and effects from the surgery before the surgery?

IF YES

- Who were you primarily informed by?

If you now look again at the entire process of providing information about your operation, which aspects could be improved from your point of view?

- (Ask in more detail if necessary)

We have now reached the end of the interview. Thank you very much for your participation and for the time you have taken.
