# Supplementary material for: Information needs of patients undergoing bariatric surgery in Germany: a qualitative study
Source: BMC Health Serv Res. 2022 Apr 19;22:515. doi: 10.1186/s12913-022-07950-2 (PMC9017015; doi:10.1186/s12913-022-07950-2)
Supplement: Supplementary file 2 — Additional file 2: Supplement 2. Coding system patients. [file 12913_2022_7950_MOESM2_ESM.docx]

***Supplement 2: Data coding system***

| Code Groups | Codes | Rules of coding | Code specification |
| --- | --- | --- | --- |
| patient characteristics | Age | Just numbers |  |
|  | Gender | Just one word |  |
|  | Clinic | Just one word |  |
|  | Education | Just one word |  |
|  | Type of insurance | Just one word |  |
|  | surgical procedures | Just one word |  |
|  | Current weight | Just numbers and unit |  |
|  | Initial weight | Just numbers and unit |  |
|  | Weight change | Not coded but calculated | initial weight and weight at the time of the interview in context of time since surgery |
|  | Time since surgery | Just numbers and unit |  |
|  | dietary supplements | Context | y/n |
|  | Why bariatric surgery? | Context | Why and who influenced to  set things in motion |
|  | First contact with surgeon/nutritionist/general practitioner/other | Context | First contact after the general idea of surgery |
|  | Why this specific clinic? | Context |  |
|  | Diabetes | Context | y/n and context e.g. medication, type, HbA1c |
| Pre-OP procedure | - Groups sessions y/n - Support group y/n | Context |  |
|  | - Time interval between application and approval/surgery - Number of clinical appointments - Number of appointments with the surgeon - Duration of the appointments with the surgeon - Number of the appointments with the nutritionists - Duration of the appointments with the nutritionist | Just numbers and units |  |
|  | - information transfer approach | Context | Group sessions, folder/map, one-on-one conversation with surgeon/nutritionist/coordinator |
|  | - Decision for the surgical procedure |  | Refers to specific surgical procedure  Point in time, participation y/n |
|  | - Groups session content |  | Profession of Groups session leader, number of group sessions, time of group sessions, group session content |
|  | - Exchange of experience with other patients |  | Other than support groups or social media e.g. friends/roommate with prior surgery |
|  | - Pre-OP support group content |  | Support group content, cooperation with the support group y/n, support group is visited by a surgeon y/n, number of joined support group sessions, duration of support group sessions |
|  | - Family/friends |  | - Did family/friends know y/n and did they know pre/post-OP  - family/friends´ reaction  - support vs. incomprehension/fear |
|  | - Additional information sources |  | Internet, social media, books etc. |
|  | - FAQs pre-OP |  |  |
| General problems | - Patient`s emotions and fears | Context |  |
|  | - Psychotherapy |  | Need for pre/post-OP psychotherapy, costs and availability of psychotherapy |
|  | - Pre-OP problems | Context | Internal/time/organizational/logistic/private procedures |
|  | - Experience of nutritionists | Context |  |
|  | - Stigmatization | Context | Negative reaction of family/friends/social context |
| Pre-OP information | - Pre-OP dietary supplements | Context | Who? When? Which information? |
|  | - Pre-OP risk disclosure - Pre-OP different surgical procedures - Pre-OP Reimbursement of surgical costs from the insurance - Pre-OP Pros and cons of the surgery - Pre-OP drinking behavior - Pre-OP general dietary information - Pre-OP information about post-OP diet - Pre-OP information about everyday life | Context | Who? When? Which information? |
| Costs | - Costs for nutrition counseling | Context | Absolut costs, information about reimbursement, information needs |
|  | - Costs for dietary supplements - Other preoperative costs - Other postoperative costs |  |  |
| Post-OP procedure | - changes in eating behavior after surgery - changes in everyday life after surgery - pre-op Information which were helpful in the period after the surgery - Post-OP nutrition counseling - Post-OP support group content - FAQ´s post-OP | Context | Problem/situation  or  Information: Who? When? Which information? |
| Post-OP problems | - dietary problems post-op - pain post-op | Context | Dietary problems: individual problems or e.g. dumping |
|  | - complications post-op | Context | OP-related complications like bleeding, infection etc. |
| Information needs | - judgement on information needs | Context |  |
| Solutions | - Problem solutions | Context |  |
