# Supplementary material for: Information needs of patients undergoing bariatric surgery in Germany: a qualitative study
Source: BMC Health Serv Res. 2022 Apr 19;22:515. doi: 10.1186/s12913-022-07950-2 (PMC9017015; doi:10.1186/s12913-022-07950-2)
Supplement: Supplementary file 3 — Additional file 3: Supplement 3. COREQ-Checklist patients. [file 12913_2022_7950_MOESM3_ESM.docx]

Supplement 3: Consolidated criteria for reporting qualitative studies (COREQ): 32-item checklist

| **Domain 1: Research team and reflexivity** | |
| --- | --- |
| *Personal characteristics* | |
| 1. Interviewer/facilitator: Which author/s conducted the interview or focus group? | 2.1  *“One interviewer (JB), who is a nutritionist and doctoral candidate with a focus on BS and an experienced qualitative researcher, conducted the audio-recorded telephone interviews.”* |
| 2. Credentials: What were the researcher’s credentials? E.g. PhD, MD |  |
| 3. Occupation: What was their occupation at the time of the study? |  |
| 4. Gender: Was the researcher male or female? |  |
| 5. Experience and training: What experience or training did the researcher have? |  |
| *Relationship with the participants* | |
| 6. Relationship established: Was a relationship established prior to study commencement? | 2.1  *“There was no relationship between the interviewer and the participants. Participants did not know the interviewer; they only knew she was a researcher at Witten/Herdecke University.”* |
| 7. Participant knowledge of the interviewer: What did the participants know about the researcher? e.g. personal goals, reasons for doing the research | Participants get detailed information about the research goal prior to the interview (see Declarations below). The researcher who conducted the interviews, determined the date of the interview with the participants and in the course of this introduced herself as a researcher at Witten/Herdecke University.  Declarations  Ethics approval and consent to participate  *“Detailed information (background, duration of the interview and publication) about the study, along with privacy statements, was given to each participant prior to the interview.”* |
| 8. Interviewer characteristics: What characteristics were reported about the interviewer/facilitator? e.g. Bias, assumptions, reasons and interests in the research topic | 2.1  “*One interviewer (JB), who is a nutritionist and doctoral candidate with a focus on BS and an experienced qualitative researcher, conducted the audio-recorded telephone interviews.* “ |
| **Domain 2: study design** | |
| *Theoretical framework* | |
| 9. Methodological orientation and Theory What methodological orientation was stated to underpin the study? e.g. grounded theory, discourse analysis, ethnography, phenomenology, content analysis | 2.5  *“The transcribed interviews (including the pretest interview) were analyzed using qualitative content analysis [13] supported by MAXQDA software.”* |
| *Participant selection* | |
| 10. Sampling How were participants selected? e.g. purposive, convenience, consecutive, snowball | 2.2  *“Patients were contacted with several approaches. We contacted all certified competence and reference centers for BS using a list of all certified centers. This list was prepared by the German Society for General and Visceral Surgery, which certifies these centers [9]. We contacted the centers by e-mail and asked them to hand out our patient recruitment flyer to eligible patients. Furthermore, we contacted several obesity and/or BS (patient organized) support groups and asked them to either hand out the patient recruitment flyer or post it on social media (e.g., Facebook). Additionally, we used the snowball sampling technique to ask participants after each interview if they had had any contact with any other eligible patients and, if so, we asked them if they could transfer our patient information flyer.”* |
| 11. Method of approach: How were participants approached? e.g. face-to-face, telephone, mail, email Reasons? | 2.1  *“One interviewer (JB), who is a nutritionist and doctoral candidate with a focus on BS and an experienced qualitative researcher, conducted the audio-recorded telephone interviews.”* |
| 12. Sample size: How many participants were in the study? | 3  *“We conducted n=14 semi-structured interviews.”* |
| 13. Non-participation How many people refused to participate or dropped out? Reasons? | 3  *“There was no dropout or refusal of participation at any time.”* |
| Setting |  |
| 14. Setting of data collection Where was the data collected? e.g. home, clinic, workplace | Since the interviews were conducted by telephone, participants could determine their own whereabouts.  2.4  *“(…)semi-structured telephone interviews (…)”* |
| 15. Presence of non-participants Was anyone else present besides the participants and researchers? | 2.1  *“One interviewer (JB), who is a nutritionist and doctoral candidate with a focus on BS and an experienced qualitative researcher, conducted the audio-recorded telephone interviews.”* |
| 16. Description of sample What are the important characteristics of the sample? e.g. demographic data, date | Results  *Table 1: Characteristics of individual participants* |
| *Data collection* | |
| 17. Interview guide Were questions, prompts, guides provided by the authors? Was it pilot tested? | 2.3  “*The interview guide (Supplement 1: interview guide) was designed prior to the interviews and consists of four main sections (demographic information, preoperative healthcare provision, postoperative healthcare provision, and information needs) with predominantly open-end questions. It was reviewed and modified by an experienced nutritionist who worked in a clinic for BS in a university hospital for many years and was the head of their nutrition team. The first interview was used as a pretest, but it resulted in no modifications of the interview guide*.” |
| 18. Repeat interviews:  Were repeat interviews carried out? If yes, how many? | There were no interviews repeated.  3  “*There was no dropout or refusal of participation at any time.”* |
| 19. Audio/visual recording:  Did the research use audio or visual recording to collect the data? | 2.1  *“One interviewer (JB), who is a nutritionist and doctoral candidate with a focus on BS and an experienced qualitative researcher, conducted the audio-recorded telephone interviews.”* |
| 20. Field notes:  Were field notes made during and/or after the interview or focus group? | NA  Due to audio recording field notes were dispensable. |
| 21. Duration:  What was the duration of the interviews or focus group? | Results  “*The duration of the interviews ranged from 21 to 70 minutes, with a mean time of 44 minutes.”* |
| 22. Data saturation:  Was data saturation discussed? | 2.2  “*Recruitment ended when saturation [10] was reached.”*  Limitations  *“A limitation of this study is the small sample size. However, we stopped recruiting in the event of suspected saturation.”* |
| 23. Transcripts returned Were transcripts returned to participants for comment and/or correction? | 2.4  “*Afterwards the researcher checked the transcripts. Participants were not involved in data processing nor analysis.* |
| **Domain 3: analysis and findings** | |
| *Data analysis* | |
| 24. Number of data coders:  How many data coders coded the data? | 2.5  “*Two researchers (JB and NK) independently analyzed one-third of the interviews with the predetermined data codes. After discussion and consensus, the data codes were modified, and the given codes were adjusted. After achieving reasonable interrater reliability, further analysis was conducted by one researcher (JB).”* |
| 25. Description of the coding tree:  Did authors provide a description of the coding tree? | 2.4/Supplement 2 data coding system  “*Furthermore, rules of coding (e.g., just one word or context) and code specifications were defined for each code and subcode (Supplement 2: data coding system).”* |
| 26. Derivation of themes:  Were themes identified in advance or derived from the data? | 2.4  “*Based on the interview guideline, data codes were developed prior to the interview analysis by one researcher (JB) and checked by another (NK). The data codes were divided into nine groups.”*  2.5  “*Two researchers (JB and NK) independently analyzed one-third of the interviews with the predetermined data codes. After discussion and consensus, the data codes were modified, and the given codes were adjusted. After achieving reasonable interrater reliability, further analysis was conducted by one researcher (JB).”* |
| 27. Software:  What software, if applicable, was used to manage the data? | 2.5  “*The transcribed interviews (including the pretest interview) were analyzed using qualitative content analysis [13] supported by MAXQDA software.”* |
| 28. Participant checking:  Did participants provide feedback on the findings? | 2.4  “*Afterwards the researcher checked the transcripts. Participants were not involved in data processing nor analysis.”* |
| *Reporting* | |
| 29. Quotations presented:  Were participant quotations presented to illustrate the themes / findings? Was each quotation identified? e.g. participant number | Quotations were illustrated in 3.2.2. and 3.3.1 with participant ID.  E.g.:  *P09: “And all the statics of my body change due to this rapid decrease - quite clearly, the whole body changes. For example, you don't think about it beforehand, you don't know. That's what you learn in the support group. That's not bad. A support group is good. I also find it useful, for example, for information about which medications you take or which dietary supplements you take.”* |
| 30. Data and findings consistent:  Was there consistency between the data presented and the findings? | 3 Results 3.1 Information provision  3.2 Information provision approaches  3.2.1 Healthcare professionals  3.2.2 Support groups  3.3 Information needs  3.3.1 General and specific information  3.3.2 Barriers to seeking information  4. Discussion 4.1 Healthcare professionals 4.2 General and specific information 4.3 Information provision: the role of support groups and digital solutions |
| 31. Clarity of major themes:  Were major themes clearly presented in the findings? | Major themes were highlighted by the layout in the results section and numbering.  3 Results 3.1 Information provision  3.2 Information provision approaches  3.2.1 Healthcare professionals  3.2.2 Support groups  3.3 Information needs  3.3.1 General and specific information  3.3.2 Barriers to seeking information |
| 32. Clarity of minor themes:  Is there a description of diverse cases or discussion of minor themes? | Not applicable |
